# VO_2_-dispersed glass: A new class of phase change material

**DOI:** 10.1038/s41598-018-20519-6

**Published:** 2018-02-02

**Authors:** Kei Muramoto, Yoshihiro Takahashi, Nobuaki Terakado, Yoshiki Yamazaki, Shigeru Suzuki, Takumi Fujiwara

**Affiliations:** 1 Department of Applied Physics, Tohoku University, 6-6-05 Aoba, Aoba-ku, Sendai 980-8579 Japan; 2 Institute of Multidisciplinary Research for Advanced Materials, Tohoku University, 2-1-1 Katahira, Aoba-ku, Sendai 980-8577 Japan

## Abstract

Energy storage technology is crucial for a sustainable society, and its realisation strongly depends on the development of materials. Oxide glass exhibits high durability. Moreover, the amorphous structure of the glass without periodic ordering demonstrates excellent formability and controllability, thus enabling a large-scale production. These factors provide impetus for the development of new materials for thermal management applications. As vanadium dioxide (VO_2_) with a strongly correlated electron system exhibits a structural phase transition, leading to a large heat of transition. Therefore, VO_2_ demonstrates immense potential as a phase change material (PCM). This study reports the fabrication of VO_2_-dispersed glass and examines its potential as a new latent heat storage material, which can be applied for massive PCM heat storage applications.

## Introduction

Heat is known to be a considerable form of energy available in the surrounding environment, e.g., solar-thermal energy and exhaust heat, which are generated by natural and artificial actions, respectively. Thus, heat is a primitive but a crucial form of energy in daily life. Hence, heat storage technology is indispensable for efficient energy utilization and sustainable development. This technology can appropriately balance the supply and demand amounts of thermal energy for different times and/or spaces, i.e., heat compensation by the shift in the time or space^1^. Thus, heat storage materials are crucial for realizing the time-/space shift in motor vehicles and solar-thermal electric generation. In particular, as the time-/space shift permits the storage of heat during the day and radiates the heat during the night, its use is also expected for the retention of temperature in a severe area experiencing a large temperature difference, e.g., an extra-terrestrial space or a planet. Particularly, in the candidate places for migration, which are accessible from the earth, i.e., the moon and Mars, the temperature-difference between the day and night is quite large, and the nighttime is extremely cold. This leads us to consider that a huge electricity is needed to keep the temperature adequate for our existence in the dwelling area and space colony. If the stored solar thermal energy is released in the nighttime, a sharp reduction of the electricity is expected. Thus, heat storage material, which has a good productivity and is expandable, are quite attractive.

**Figure 1:**
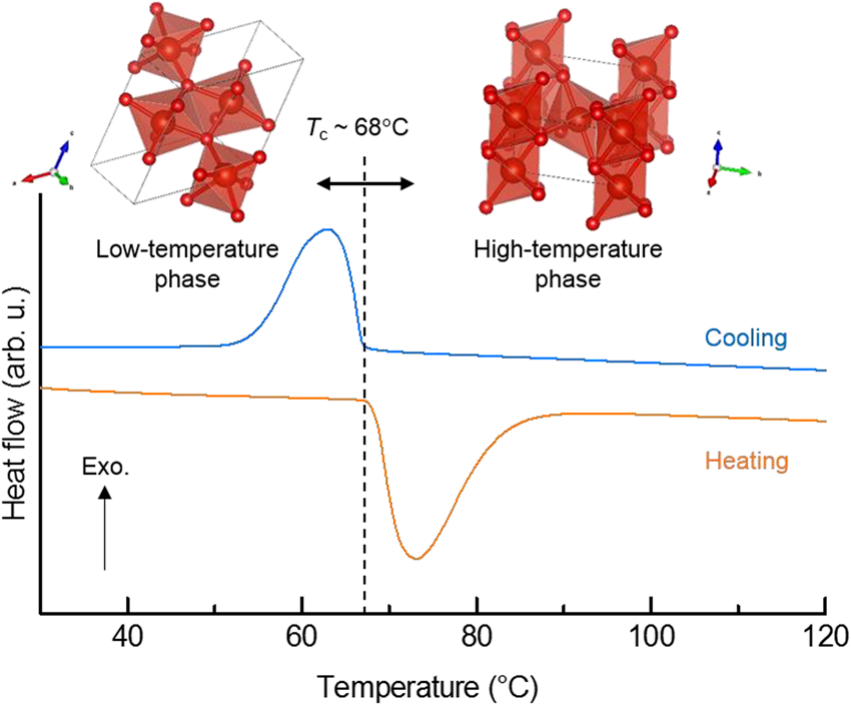
Endothermic or exothermic reaction of the VO_2_ phase caused by phase transition. DTA measurement was carried out using reagent-grade VO_2_ powder. In the DTA curves, endothermic and exothermic peaks were observed in the heating and cooling processes, respectively, corresponding to the phase transition between the monoclinic and tetragonal (rutile-type) structures at a *T*_c_ of ~68 °C.

Latent heat storage is based on the capture or release of energy when a material undergoes a phase change from, for example, solid to liquid or vice versa. Note that heat storage materials based on this phase transition are referred to as phase change materials (PCMs)^2–6^. PCMs can accumulate thermal energy, which exhibits intermittent characteristics, and subsequently generate heat at a constant (phase-transition) temperature related to the latent heat. For example, ice, paraffin, fatty acids, and inorganic hydrates are well-known PCMs that can store thermal energy at low temperatures (<150 °C)^7^. The heat storage mechanism of these PCMs involves a solid–liquid phase transition; hence, it is imperative to ensure that the PCM inside the container is maintained in the liquid state. Moreover, some hazards are inevitable, i.e., damage to the container because of the large volume change occurring during the phase transition and leaking out of the liquid. In these circumstances, it is of particular interest in developing new candidates for novel heat storage material, e.g., λ-trititanium pentoxide (Ti_3_O_5_) based solid–solid phase transition, microencapsulated metal-based PCM, and so on^7,8^: Vanadium dioxide (VO_2_) exhibits a strongly correlated electron system, which exhibits a structural phase transition at 68 °C. A reversible change is observed between the monoclinic (low-temperature phase) and tetragonal (high-temperature phase) crystal structures accompanied with an exothermic or endothermic reaction (Fig. 1)^9–12^. Hence, the above-mentioned hazards can be avoided. In addition, the latent heat of VO_2_ (~237 J/cm^3^) is comparable to that of the PCMs reported thus far^13^, making it adequate for practical applications. Recently, VO_2_ powder for PCM has been developed and is commercially-available from the companies; Kojundo chemical laboratory Co., Ltd. (Smartec^®^ HS) and NIPPON DENKO CO., LTD. However, as VO_2_ is available as a powder, it is mandatory to use a container if the VO_2_ powder is used as the PCM. In addition, vanadium is a rare metal; hence, VO_2_ reagents are expensive. Thus, VO_2_ exhibits some disadvantages that need to be improved so as to enable large-scale PCM applications.

**Figure 2:**
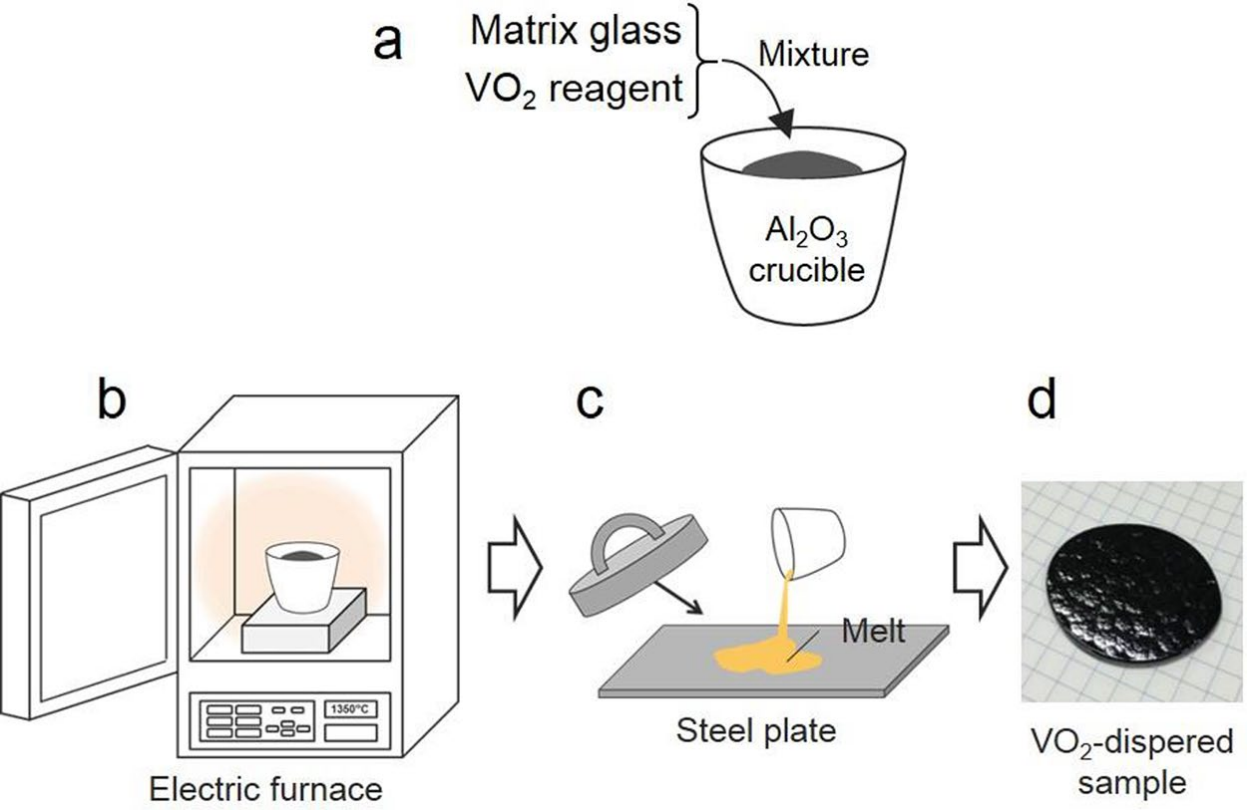
Incorporation method for the preparation of the VO_2_-dispersed glass. (**a**) The powdered glass matrix and VO_2_ powder were mixed, and the mixture was subsequently added into a crucible with a lid. (**b**) The mixture was melted in an electric furnace. (**c**) The melts were poured onto a steel plate and were quenched by another steel plate. (**d**) The VO_2_-dispersed glass was obtained.

In this study, a new PCM is proposed, i.e., VO_2_-dispersed glass, where the VO_2_ powders embedded in the glass matrix, serves as a durable container-free PCM and prevents the degradation of the VO_2_ phase because of oxidation and moisture. Glass exhibits immense advantages from the viewpoint of materials science, e.g., large-scale or mass production, flexibility, and formability. Hence, recently, glass has been extensively examined not only for photonics but also as energy-related materials^14–17^. Based on this background, VO_2_-dispersed glass exhibits immense potential for PCM on the basis of the solid–solid phase transition. An incorporation method^18^ is utilised for fabricating the dispersed glass (Fig. 2), aiming to realise the all-solid PCM.

## Results

### Selection of the glass matrix composition

First, several glasses were prepared to investigate the composition of the glass matrix suitable for the VO_2_ dispersion. In this study, three compositions as candidates for the glass matrix, i.e., 35BaO–65B_2_O_3_, 15B_2_O_3_–10P_2_O_5_–75V_2_O_5_, and 30BaO–10TeO_2_–60V_2_O_5_, were examined. A borate system was selected because of a previously reported study in which vanadate compounds have been embedded in a borate glass matrix^19^. Vanadate systems have been selected because a low-processing temperature is expected for synthesis^20–22^.

**Figure 3:**
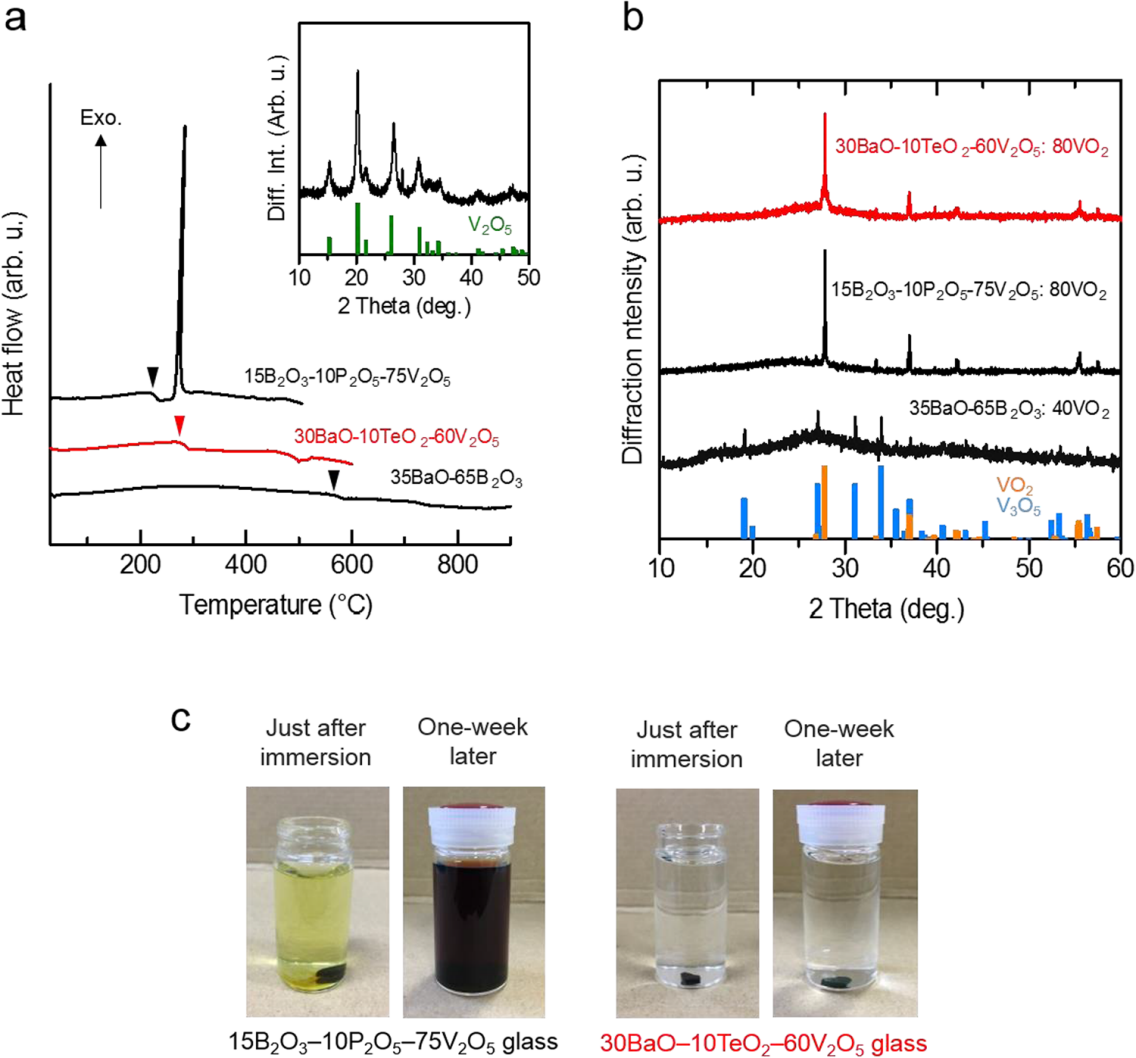
Preliminary assessment of the glass matrix. (**a**) DTA results of 35BaO–65B_2_O_3_, 15B_2_O_3_–10P_2_O_5_–75V_2_O_5_, and 30BaO–10TeO_2_–60V_2_O_5_ glasses in the bulk form, which were selected as candidate matrices for the VO_2_ dispersion. Inverted triangles indicate the glass-transition temperatures, namely, *T*_g_ = 580 °C, 225 °C, and 276 °C for the BaO–B_2_O_3_, B_2_O_3_–P_2_O_5_–V_2_O_5_, and BaO–TeO_2_–V_2_O_5_ systems, respectively. In addition, the inset shows the powder XRD pattern of the B_2_O_3_–P_2_O_5_–V_2_O_5_ sample heat-treated at a crystallisation peak temperature, i.e., *T*_p_ = 279 °C, in addition to the pattern based on the ICDD of V_2_O_5_ (#41-1426). (**b**) Powder XRD patterns of the candidate glasses dispersed with VO_2_ powder on the basis of the incorporation method (see Method). The values at the end of glass composition represent the additive amount of dispersed VO_2_ (in mol%). In addition, the ICDD patterns of VO_2_ (#43-1051) and V_3_O_5_ (#72-0977) are included. (**c**) The immersion test for water resistivity in the V_2_O_5_-based matrix glass.

Differential thermal analysis (DTA) (Fig. 3(a)) revealed that the BaO–B_2_O_3_ and BaO–TeO_2_–V_2_O_5_ systems exhibit high thermal stability against crystallisation because the exothermic peak corresponding to the amorphous–crystal phase transformation was absent, whereas a strong, sharp crystallisation peak was observed for the B_2_O_3_–P_2_O_5_–V_2_O_5_ system, corresponding to formation of the V_2_O_5_ phase (inset). On the other hand, vanadate systems exhibited a considerably lower glass-transition temperature (*T*_g_) compared with that of the borate system. A low *T*_g_ is preferable for the VO_2_ dispersion to proceed at a low temperature with respect to energy savings as well as the prevention of degradation of VO_2_ because of high-temperature exposure.

Next, the incorporation method was carried out as a trial to fabricate VO_2_-dispersed glasses, which were subsequently analysed by powder X-ray diffraction (XRD) (Fig. 3(b)). The VO_2_ phase in the borate system was transformed into V_3_O_5_ (or the so-called Magneli phase)^23^, and the size of the Magneli phase (~10 μm, Supplementary Fig. S2) is less than that of the VO_2_ reagent (~20 μm, Supplementary Fig. S1), suggesting the elution or diffusion of vanadium into the BaO–B_2_O_3_ glass matrix. On the other hand, the VO_2_ phase was stably retained in case of the vanadate systems. This result led us to expect that vanadate systems are suitable for fabricating VO_2_-dispersed samples. In addition, the water stability test was carried out using the candidate vanadate system glasses. After the immersion of the B_2_O_3_–P_2_O_5_–V_2_O_5_ system glass into water, a pale-yellow colour was immediately observed, and then the water became black after 1 week (Fig. 3(c)). Meanwhile, the BaO–TeO_2_–V_2_O_5_ system remained transparent, indicative of its high resistively against water or moisture. Thus, the BaO–TeO_2_–V_2_O_5_ system demonstrates immense potential as glass matrices, which can be used for the VO_2_-dispersed PCM. Hence, 30BaO–10TeO_2_–60V_2_O_5_ is selected as the glass matrix.

### Characterisation of the VO_2_-dispersed glass

**Figure 4:**
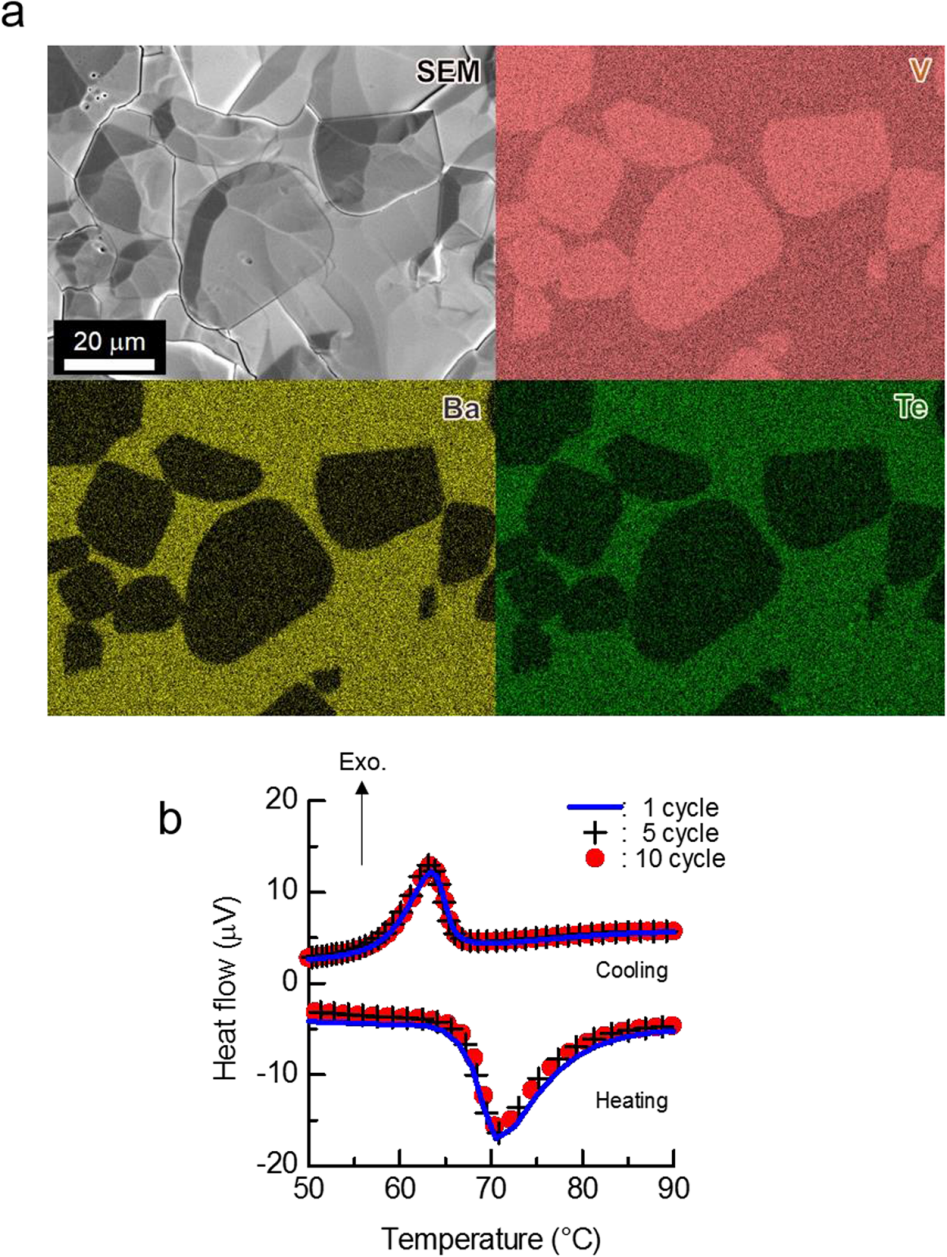
Characteristics of the VO_2_-dispersed sample in 30BaO–10TeO_2_–60V_2_O_5_: 80VO_2_. (**a**) SEM (secondary electron) and the elemental mapping results of the sample with *x* = 80. Scale bar corresponds to 20 um. (**b**) DTA results in cooling-/heating-cyclic condition at 1-, 5- and 10-cycles (heating and cooling rate: 10 K/min; sample weight: ~40 mg).

VO_2_-dispered glasses with different additive amounts (*x*; mol%) of VO_2_ powder, i.e., 30BaO–10TeO_2_–60V_2_O_5_:*x*VO_2_ composites, were fabricated according to the protocol described in the Method section. Scanning electron microscopy (SEM) analyses revealed that the incorporated VO_2_ powders are stably embedded in the matrix, with no significant aggregation of the powders (*x* = 80; Fig. 4(a)). Elemental mapping results revealed a clear boundary between the VO_2_ particles and glass phases. In addition, the migration of barium (Ba) and tellurium (Te) surrounding the VO_2_ particles and the diffusion of vanadium (V) into the glass matrix were barely observed. To primarily examine the heat storage property, thermal cycling test was also carried out by means of DTA in the VO_2_-dispersed sample. Clear endothermic and exothermic peaks were observed in the temperature range of ~60–80 °C, which are attributed to the phase-transition of VO_2_, during the heating and cooling process, respectively. (Fig. 4(b)). The peaks of 10-cycle could be almost superimposed on that of 1-cycle. In addition, the SEM observation revealed no significant change in the microstructure for the sample after the cycling test (Supplementary Fig. S3). Since any collapses were hardly observed in the sample subjected to the cycling test, it is suggested that the dispersed glass possesses a high thermal repeatability.

### Thermal properties

**Figure 5:**
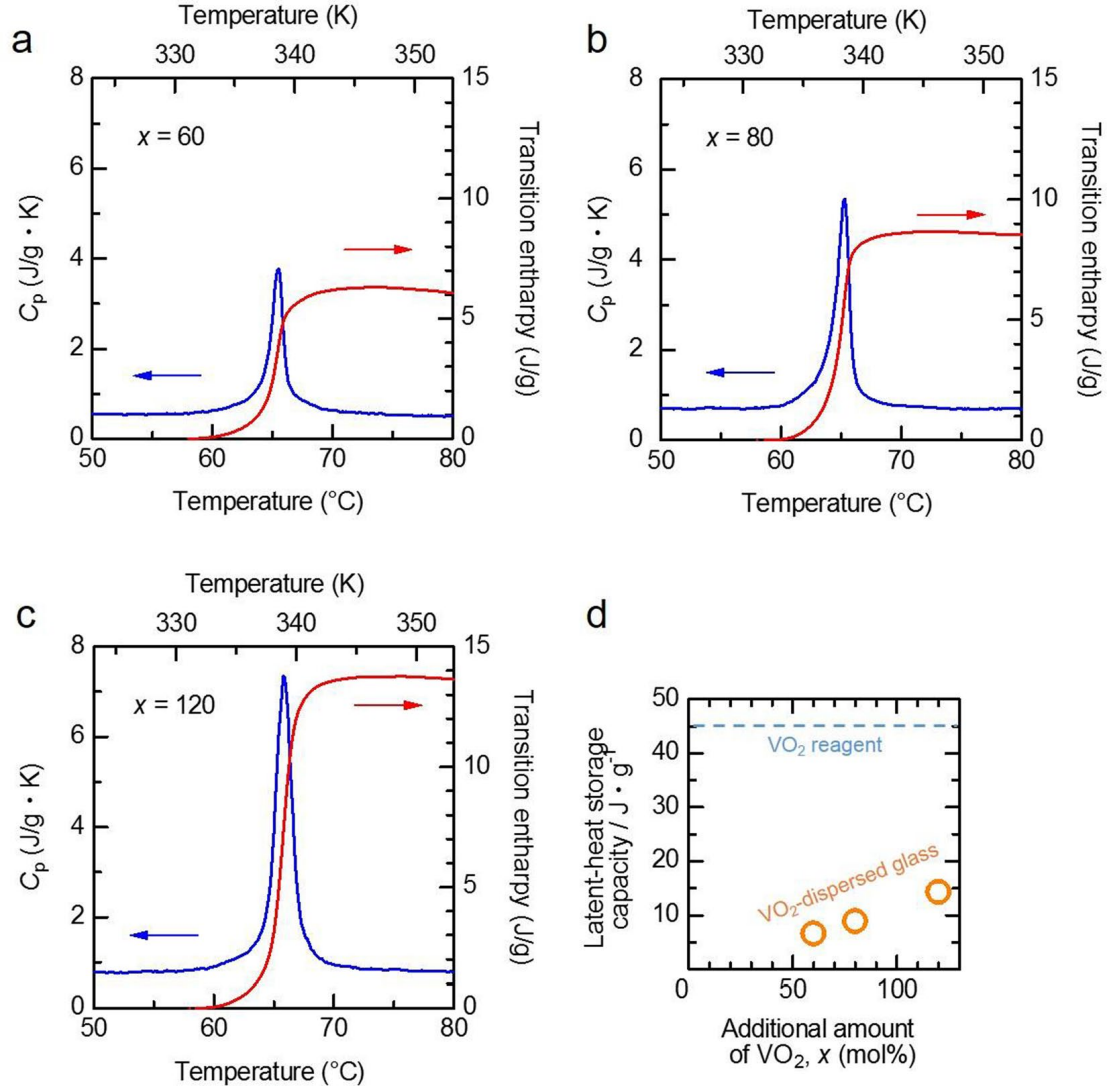
Thermal properties of the VO_2_-dispersed samples. (**a**) Heat capacity and transition enthalpy for the VO_2_-dispersed glasses in the BaO–TeO_2_–V_2_O_5_ system with *x* = 60, (**b**) 80, and (**c**) 120. The transition enthalpy, Δ*H*, was obtained by thermodynamic treatment based on $\triangle H\,=\int {C}_{p}{\rm{d}}T$ because the enthalpy corresponded to the latent heat storage capacity. (**d**) The latent heat storage capacity as a function of *x*.

**Figure 6:**
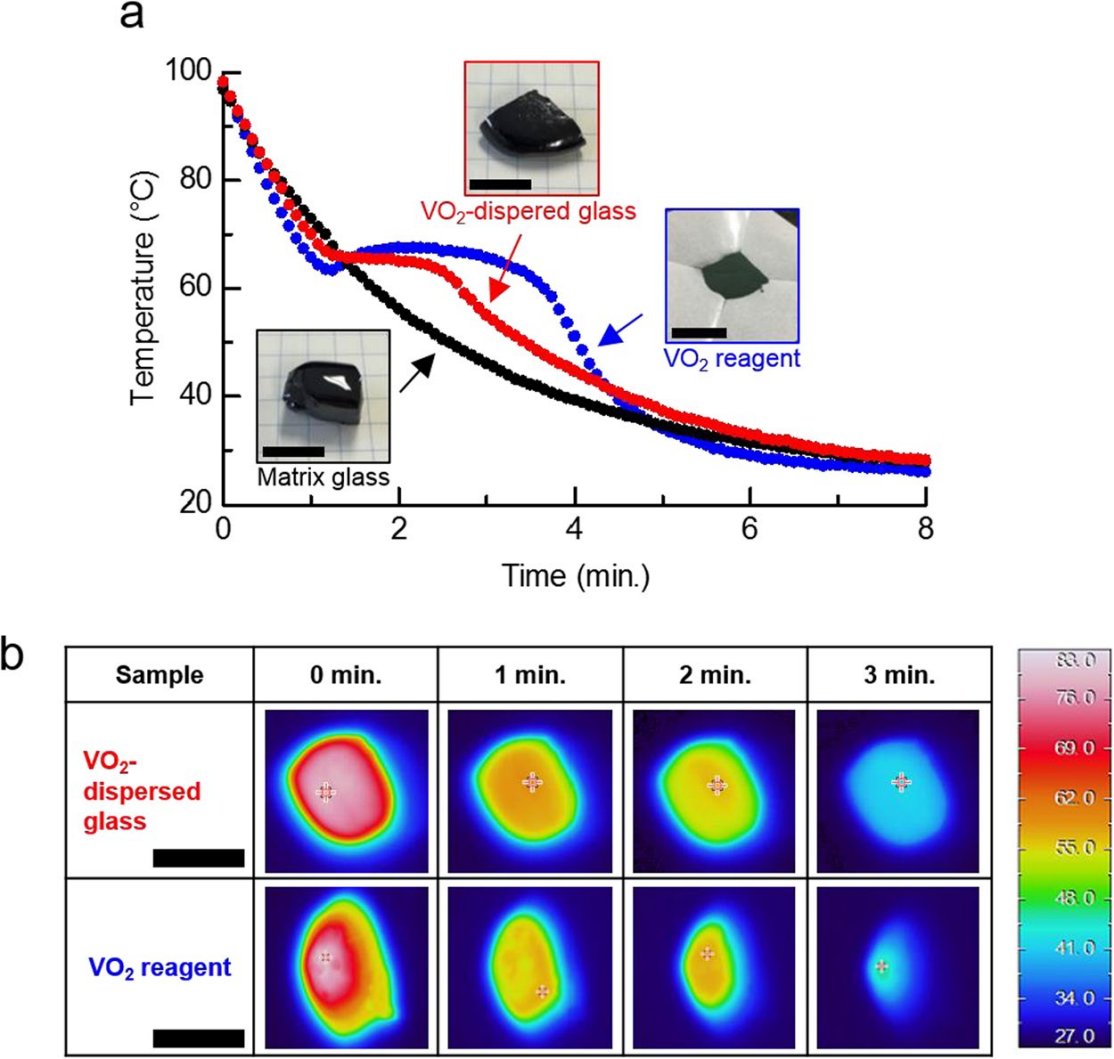
Temperature retention of the examined samples. (**a**) Temperature change of the matrix glass in BaO–TeO_2_–V_2_O_5_ (bulk; ~2.4 g), VO_2_ (powder; ~2.3 g), and VO_2_-dipersed glass (*x* = 120, bulk; ~2.2 g) vs. time. Dispersed samples exhibited a plateau around the phase transition temperature, related to the latent heat of the incorporated VO_2_ phase. **(b)** Thermal images of the dispersed sample and VO_2_. In the both experiments of (a) and (b), temperature of the samples were risen to ~100 °C by a resistance heater, and then their surface-temperatures were monitored by use of the thermography. Black bars in (**a**) and (**b**) correspond to the scale of ~1 cm.

To examine the latent heat storage function in detail, DSC measurements were carried out. A steep change in the specific heat (peak of the *C*_p_–*T* curve), related to the phase transition of the dispersed VO_2_ powder, was observed around the phase-transition temperature of VO_2_ (*T*_c_~68 °C), and then the transition enthalpy (Δ*H*), which corresponded to the latent heat storage capacity, was estimated (Fig. 5a,b, and c) for *x* = 60, 80, and 120, respectively). The Δ*H* values increased with *x*, and finally the sample with *x* = 120 exhibited a Δ*H* value of ~14.3 J/g (Fig. 5(d)), corresponding to ca. 30% of that of the VO_2_ reagent (~45 J/g, Supplementary Fig. S1). Thus, the latent heat storage function is imparted to the glass-based material via the VO_2_ dispersion. In addition, the temperature retention property of the VO_2_-dispersed glass was assessed. Small pieces of matrix glass (free from VO_2_, bulk state) and dispersed glass (*x* = 120, bulk) and VO_2_ reagent (powder), with similar weight (~2.2–2.4 g), were heated at around ~100 °C, and then were left at room temperature (RT). Variations in the surface temperature were monitored by thermography. In the matrix glass, the temperature monotonically decreased with time, eventually returning to RT (Fig. 6a)). On the other hand, the VO_2_ reagent exhibited a temperature plateau for ~2.5 min, corresponding to the latent heat related to the phase transition, with subsequent cooling to RT. In addition, the dispersed glass exhibited a plateau for ~1.5 min, indicative of the temperature retention properties. Thermographic images also aided in the better understanding of the temperature variation. The dispersed glass and VO_2_ reagent exhibited similar temperature distribution after ~1 min (start of the plateau) (Fig. 6(b)). Notably, the VO_2_ reagent exhibited a fluctuation in the image colour, indicative of a non-homogeneous temperature gradient. This fluctuation was related to the difference in the packing density of the VO_2_ powder. However, the dispersed glass barely exhibited any fluctuation.

## Discussion

V_2_O_5_ is a glass-forming oxide according to Sun’s classification^24^, and is easily vitrified by addition of network-modifiers^25^. V_2_O_5_ is the major component in the examined B_2_O_3_–P_2_O_5_–V_2_O_5_ and BaO–TeO_2_–V_2_O_5_ glasses. Although both of these glasses exhibited low *T*_g_, their thermal stabilities against crystallisation and water resistivity were considerably different. A previous study on the glass structure in the P_2_O_5_–V_2_O_5_ system have reported that the glass network comprises layers of pyramidal VO_5_ units, which are interlinked together by PO_4_ units^26^. Because of the open network structure of glass, water molecules easily attack the PO_4_ units^27–30^, eventually destroying the glass network. Hence, the low stability and resistivity in B_2_O_3_–P_2_O_5_–V_2_O_5_ are thought to be related to a similar structural scenario. Meanwhile, the glasses in the TeO_2_–V_2_O_5_ system comprise of V–O and Te–O polyhedral units, leading to a three-dimensional network structure in binary and multicomponent systems^20–22^. Hence, it is hypothesised that a 3D structure provides a stable network and considerably contributes to the high thermal stability against crystallisation and water resistivity in the BaO–TeO_2_–V_2_O_5_ system.

The homogeneity in the temperature retention (no fluctuation of temperature distribution, Fig. 5) was observed in the dispersed glass, and the presence of the glassy phase probably contributed to the minimisation of the pores occurring between the VO_2_ particles, which necessarily occurred in the powder state. Temperature fluctuations should be enlarged via the expansion of the material in case the PCM powder is used; hence, dispersion or incorporation plays a crucial role in maintaining the homogeneity of the spatial temperature distribution. Moreover, generally, thermal conductivity (*Λ*) of multicomponent glass possesses *Λ* ~ 1 Wm^−1^K^−1^, regardless of its composition/system, and such a low *Λ* originates in prevention of phonon-propagation due to the random-network (disordered) structure without transition symmetry, e.g., 40P_2_O_5_–60V_2_O_5_ glass; *Λ* ~ 0.8 Wm^−1^K^−1^ (at 350 K or 77 °C)^31^. Although the *Λ*-value of matrix glass in this study (30BaO–10TeO_2_–60V_2_O_5_) is considered to be lower than that of VO_2_ crystal (*Λ* ~ 4 Wm^−1^K^−1^ and *Λ* ~ 5 Wm^−1^K^−1^ in monoclinic and tetragonal systems, respectively)^32^, the low *Λ*-value of glass is possibly preferable from the viewpoint of heat-retaining property because a gradual cooling is expected in the VO_2_-dispersed glass before/after temperature retention (plateau), and we can also see this trend in Fig. 6(a). The acquisition of the temperature retention properties in glass materials with the dispersion or immobilisation of the VO_2_ phase is expected to be valuable to exploit new functions and applications.

There are some reports about heat storage material consisting of polymers, e.g., polymer blend PCM. However, taking that oxide (or ceramic) material basically possess a high mechanical/thermal properties compared to polymer (organic) material into account, glass-based PCM is expected to have a long-term reliability. Furthermore, glass-based PCMs are considered to demonstrate potential for massive thermal storage applications, for example, in space development and terraforming, on the basis of the advantages of glass materials, e.g., large-scale and mass production. In particular, the presence of abundant glass and related minerals (e.g., pyroxene and olivine) on Mars^33^ has also vigorously stimulated the study of glass-based PCMs.

In conclusion, a new PCM based on glass materials, i.e., VO_2_-dispered glass in multicomponent systems, is reported, and its latent heat storage and temperature retention properties are demonstrated. On the other hand, because of the dispersed glass still being a prototype, some issues should be overcome, e.g., improvement of the heat storage amount. Nevertheless, as the technology for glass–crystal composites and their industrialisation has been previously reported^34^, the results obtained herein demonstrate significance as the first step in the development of all-solid PCMs.

## Methods

### Preparation of the matrix glass

The glass matrix compositions were 35BaO–65B_2_O_3_, 15B_2_O_3_–10P_2_O_5_–75V_2_O_5_, and 30BaO–10TeO_2_**–**60V_2_O_5_ (mol%). Commercial reagent-grade powders of BaCO_3_, B_2_O_3_, (NH_4_)_2_HPO_4_, V_2_O_5_, and TeO_2_ were used as raw materials. Glasses were prepared by a conventional melt-quenching technique using an alumina crucible with a lid. Melting conditions were as follows: 1200 °C for 30 min (BaO–B_2_O_3_ system), 800 °C for 60 min (B_2_O_3_–P_2_O_5_–V_2_O_5_), and 800 °C for 60 min (BaO–TeO_2_–V_2_O_5_) under air. The melts were poured onto a steel plate heated at ~200 °C, followed by pressing using another steel plate to obtain the as-quenched samples (Quenching rate: ~10^1^–10^2^ K/sec). Their samples were confirmed to be in the glassy state as evidenced by X-ray diffraction (XRD) analysis.

### Dispersion of VO_2_ in the glass matrix

To fabricate the VO_2_-dispersed glass, the incorporation method reported in the study by Komatsu *et al*.^18^ was utilised. Figure 2 shows the schematics of the procedure. A powdered matrix glass and VO_2_ powder (purity: 99.9%; Kojundo Chemical Laboratory Co., Ltd.) were thoroughly mixed using an alumina mortar. Second, the mixture was added into an alumina crucible and melted under the following conditions: 1200 °C for 10 min (BaO–B_2_O_3_ system), 1200 °C for 15 min (B_2_O_3_–P_2_O_5_–V_2_O_5_), and 900 °C for 10 min (BaO–TeO_2_–V_2_O_5_) under air. The quenching process was similar to that utilised during the preparation of the matrix glass, finally furnishing VO_2_-dispered glasses with different matrices.

### Characterisation of the matrix glass and VO_2_-dipersed glass

In the studied glasses and VO_2_-dispersed samples, the state of the material and crystals were observed by XRD (Cu-Kα radiation). Microscopic observation was carried out by scanning electron microscopy (SEM) equipped with energy-dispersive X-ray spectroscopy. Water stability was examined by the immersion of the samples (~0.2 g; bulk form) into water.

The thermal properties of the matrix and VO_2_-dispersed glasses were examined by differential thermal analysis (DTA; heating rate of 10 K/min, Rigaku Corporation, Thermoplus TG8120). The transition enthalpy (Δ*H*), corresponding to the amount of the stored latent heat, of the VO_2_-dispersed samples was evaluated by differential scanning calorimetry (DSC; heating rate of 1 K/min, Seiko Instruments Inc., DSC6220). The specific heat *C*_p_ [$={(\partial H/\partial T)}_{p}$] was measured as a function of temperature (*T*), and Δ*H* was estimated on the basis of the thermodynamic relation: $\triangle H\,=\int {C}_{p}{\rm{d}}T$. The sample state used in the measurement was the bulk form with a weight of ca. 10 mg. The time dependence of temperature in the examined samples was evaluated by a thermography test (Nippon Avionics Co., Ltd.; R300SR-S).

## Electronic supplementary material

Supplementary Information
