# Supplementary material for: VO2-dispersed glass: A new class of phase change material
Source: Sci Rep. 2018 Feb 2;8:2275. doi: 10.1038/s41598-018-20519-6 (PMC5797218; doi:10.1038/s41598-018-20519-6)
Supplement: Supplementary file 1 — Supplementary Information [file 41598_2018_20519_MOESM1_ESM.doc]

**VO_2_-dispersed glass: A new class of phase change material**

Kei Muramoto^1^, Yoshihiro Takahashi^1,†^, Nobuaki Terakado^1^, Yoshiki Yamazaki^2^, Shigeru Suzuki^2^, and Takumi Fujiwara^1,‡^

^1^ *Department of Applied Physics, Tohoku University, 6-6-05 Aoba, Aoba-ku, Sendai 980-8579, Japan*

^2^ *Institute of Multidisciplinary Research for Advanced Materials, Tohoku University, 2-1-1 Katahira, Aoba-ku, Sendai 980-8577, Japan*

**Supplementary Information**


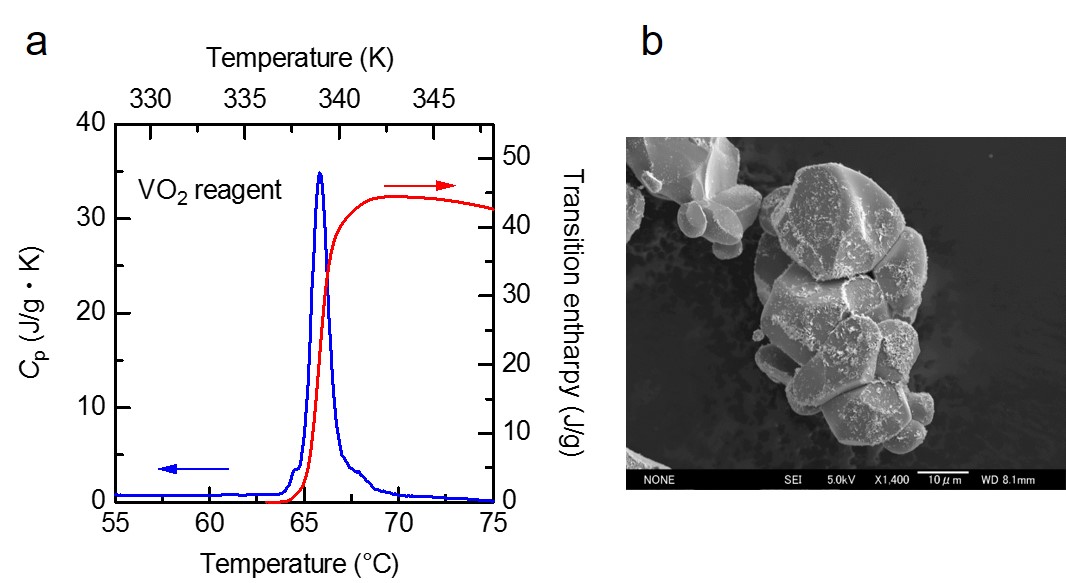


**Figure S1. Several features in the VO_2_ powder.** (**a**) Heat capacity and transition enthalpy in the VO_2_ reagent, which is commercially-available (Kojundo chemical laboratory Co., Ltd.). The thermal properties were studied on the basis of DSC measurement. The measurement revealed that the transition enthalpy of VO_2_ reagent can be evaluated to be Δ*H*~45 J/g. (**b**) SEM result of the VO_2_ reagent (secondary-electron image). The particle size was approximately 20 μm.


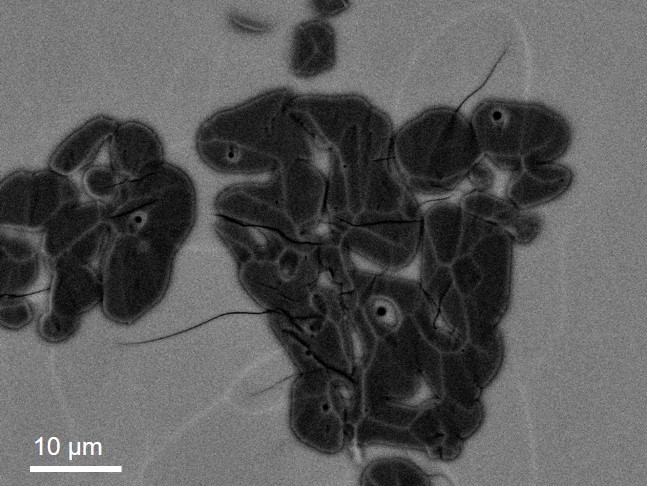


**Figure S2. Internal texture of the VO_2_-dispersed sample in borate system.** The SEM result corresponds to a backscattered-electron image in the sample that 40 mol% of the VO_2_ was added into the 35BaO–65B_2_O_3_ glass (i.e., 35BaO–65B_2_O_3_: 40VO_2_). The added VO_2_ phase was transformed into V_3_O_5_ (Magneli phase; cf. Fig. 3(b)). The particle size of Magneli phase was approximately 10 μm.


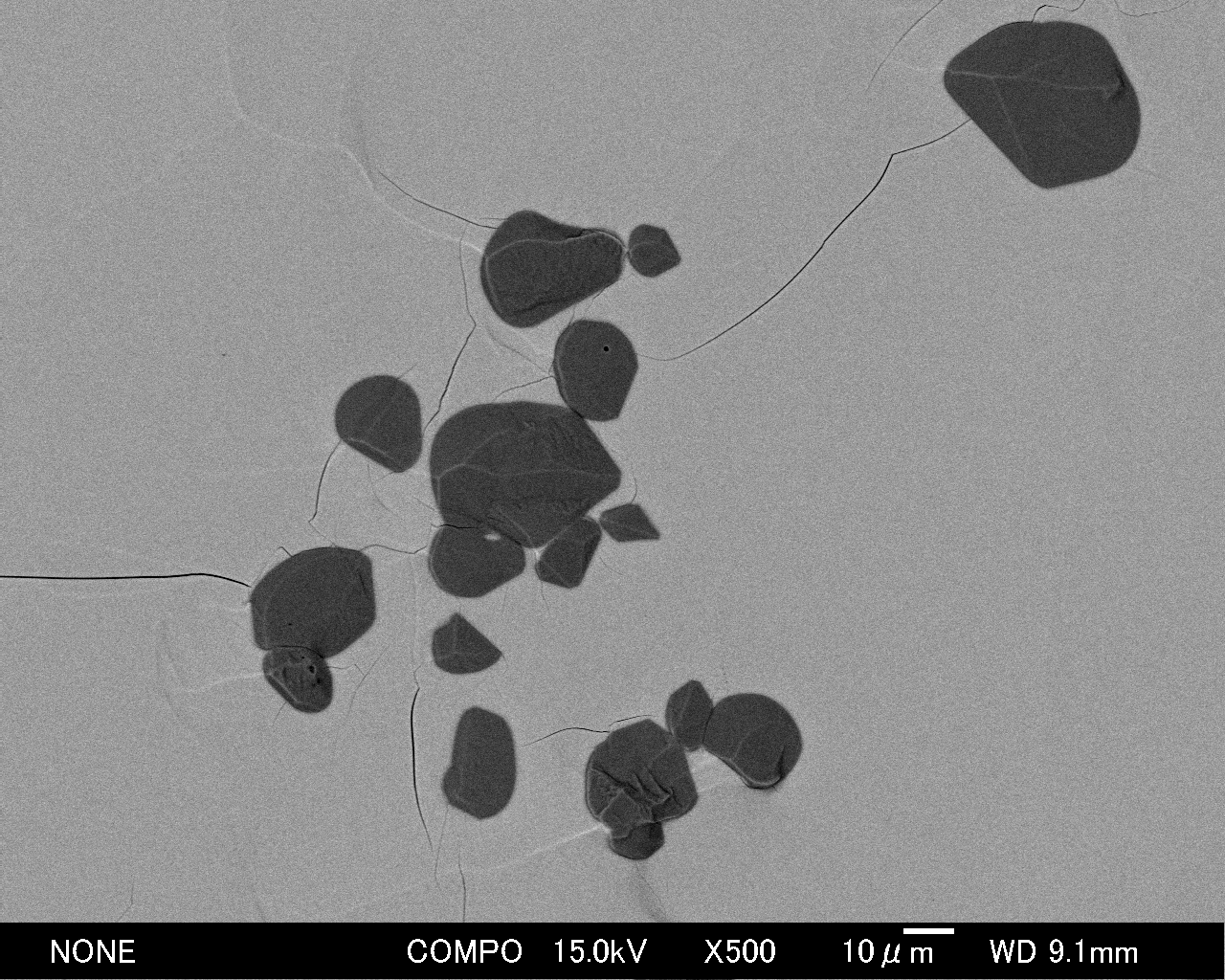


**Figure S3. Internal texture of the VO_2_-dispersed sample after thermal cycling test in BaO‒TeO_2_‒V_2_O_5_ system.** The SEM result corresponds to a backscattered-electron image in the sample that 80 mol% of the VO_2_ was added into the 30BaO‒10TeO_2_‒60V_2_O_5_ glass (i.e., 30BaO‒10TeO_2_‒60V_2_O_5_: 80VO_2_), which was subjected to cooling-/heating-cyclic processes (~50‒100°C; 10 times).
